# 2-Phenyl-1*H*-imidazol-3-ium hydrogen oxalate

**DOI:** 10.1107/S1600536811023300

**Published:** 2011-06-22

**Authors:** Jin-Na Song

**Affiliations:** a School of Biological and Agricultural Engineering, Jilin University, Changchun 130022, People’s Republic of China

## Abstract

In the title mol­ecular salt, C_9_H_9_N_2_^+^·C_2_HO_4_^−^, the dihedral angle between the aromatic rings of the cation is 17.5 (3)° and the dihedral angle between the –CO_2_H and –CO_2_ groups of the anion is 38.6 (2)°. In the crystal, the components inter­act by way of O—H⋯O and N—H⋯O hydrogen bonds.

## Related literature

For backgrond to 2-phenyl­imidazole as a ligand, see: Liu *et al.* (2008 ▶). For a related 2-phenyl­imidazolium nitrate structure, see: Zhang *et al.* (2007 ▶).
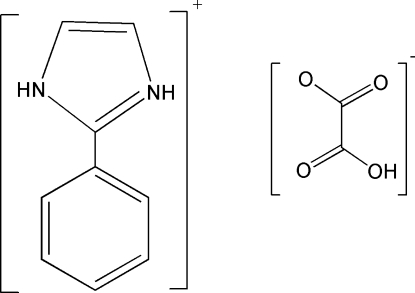


## Experimental

### 

#### Crystal data

C_9_H_9_N_2_^+^·C_2_HO_4_^−^
*M* *_r_* = 234.21
Triclinic, 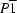

*a* = 5.571 (4) Å
*b* = 9.216 (5) Å
*c* = 11.918 (6) Å
α = 70.262 (5)°
β = 80.460 (1)°
γ = 74.871 (5)°
*V* = 554.0 (6) Å^3^
*Z* = 2
Mo *K*α radiation
μ = 0.11 mm^−1^
*T* = 293 K
0.22 × 0.20 × 0.15 mm

#### Data collection

Oxford Diffraction Gemini R Ultra CCD diffractometer
Absorption correction: multi-scan (*CrysAlis RED*; Oxford Diffraction, 2006 ▶) *T*_min_ = 0.38, *T*_max_ = 0.57
4030 measured reflections
2505 independent reflections
1629 reflections with *I* > 2σ(*I*)
*R*_int_ = 0.031

#### Refinement

*R*[*F* ^2^ > 2σ(*F* ^2^)] = 0.044
*wR*(*F* ^2^) = 0.104
*S* = 0.93
2505 reflections
154 parameters
H-atom parameters constrained
Δρ_max_ = 0.20 e Å^−3^
Δρ_min_ = −0.38 e Å^−3^

### 

Data collection: *CrysAlis CCD* (Oxford Diffraction, 2006 ▶); cell refinement: *CrysAlis RED* (Oxford Diffraction, 2006 ▶); data reduction: *CrysAlis RED*; program(s) used to solve structure: *SHELXS97* (Sheldrick, 2008 ▶); program(s) used to refine structure: *SHELXL97* (Sheldrick, 2008 ▶); molecular graphics: *SHELXTL* (Sheldrick, 2008 ▶); software used to prepare material for publication: *SHELXTL*.

## Supplementary Material

Crystal structure: contains datablock(s) global, I. DOI: 10.1107/S1600536811023300/hb5908sup1.cif

Structure factors: contains datablock(s) I. DOI: 10.1107/S1600536811023300/hb5908Isup2.hkl

Supplementary material file. DOI: 10.1107/S1600536811023300/hb5908Isup3.cml

Additional supplementary materials:  crystallographic information; 3D view; checkCIF report

## Figures and Tables

**Table 1:** Hydrogen-bond geometry (Å, °)

*D*—H⋯*A*	*D*—H	H⋯*A*	*D*⋯*A*	*D*—H⋯*A*
O2—H2*A*⋯O4^i^	0.82	1.76	2.5793 (19)	172
N2—H2⋯O3^ii^	0.86	1.90	2.732 (2)	164
N1—H1⋯O4^i^	0.86	1.93	2.777 (2)	169

Symmetry codes: (i) 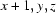; (ii) 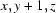.

**Fig. 1.**
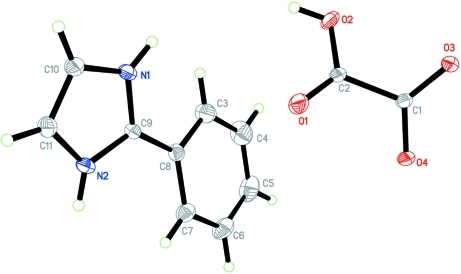
The structure of (I), showing displacement ellipsoids drawn at the 30% probability level.

## supplementary crystallographic information

### Comment

2-Phenylimidazole has been extensively used to build supramolecular architectures because of its excellent coordinating abilities and fruitful aromatic systems, (Liu *et al.*, 2008). The 2-phenylimidazolium nitrate structure has been reported as a hemihydrate (Zhang *et al.*, 2007). In this work, we will report the synthesis and crystal structure of the 2-phenylimidazolium hydrogen oxalate, namely, C_11_H_10_N_2_O_4_.

The asymmetric unit of the title compound contains one 2-phenylimidazolium cation and one hydrogen oxalate anion (Fig. 1). There are O—H···O and N—H···O hydrogen-bonding interactions in the structure (Table I).

### Experimental

A mixture of 2-phenylimidazole (0.3 mmol), oxalic acid (0.3 mmol) and H_2_O (8 ml) was mixed. After one week, colorless blocks of the title compound were obtained at room temperature.

### Refinement

All H atoms on C and N atoms were positioned geometrically (N—H = 0.86 Å, O—H = 0.82 Å and C—H = 0.93 Å) and refined as riding, with *U*_iso_(H)=1.2*U*_eq_(carrier).

### Crystal data

**Table:**

C_9_H_9_N_2_^+^·C_2_HO_4_^−^	*Z* = 2
*M**_r_* = 234.21	*F*(000) = 244
Triclinic, *P*1	*D*_x_ = 1.404 Mg m^−^^3^
Hall symbol: -P 1	Mo *K*α radiation, λ = 0.71073 Å
*a* = 5.571 (4) Å	Cell parameters from 2505 reflections
*b* = 9.216 (5) Å	θ = 1.8–29.1°
*c* = 11.918 (6) Å	µ = 0.11 mm^−^^1^
α = 70.262 (5)°	*T* = 293 K
β = 80.460 (1)°	Block, colorless
γ = 74.871 (5)°	0.22 × 0.20 × 0.15 mm
*V* = 554.0 (6) Å^3^	

### Data collection

**Table:**

Oxford Diffraction Gemini R Ultra CCD diffractometer	2505 independent reflections
Radiation source: fine-focus sealed tube	1629 reflections with *I* > 2σ(*I*)
graphite	*R*_int_ = 0.031
Detector resolution: 10.0 pixels mm^-1^	θ_max_ = 29.1°, θ_min_ = 1.8°
ω scans	*h* = −5→7
Absorption correction: multi-scan (*CrysAlis RED*; Oxford Diffraction, 2006)	*k* = −9→11
*T*_min_ = 0.38, *T*_max_ = 0.57	*l* = −14→16
4030 measured reflections	

### Refinement

**Table:**

Refinement on *F*^2^	Primary atom site location: structure-invariant direct methods
Least-squares matrix: full	Secondary atom site location: difference Fourier map
*R*[*F*^2^ > 2σ(*F*^2^)] = 0.044	Hydrogen site location: inferred from neighbouring sites
*wR*(*F*^2^) = 0.104	H-atom parameters constrained
*S* = 0.93	*w* = 1/[σ^2^(*F*_o_^2^) + (0.0587*P*)^2^] where *P* = (*F*_o_^2^ + 2*F*_c_^2^)/3
2505 reflections	(Δ/σ)_max_ < 0.001
154 parameters	Δρ_max_ = 0.20 e Å^−^^3^
0 restraints	Δρ_min_ = −0.38 e Å^−^^3^

### Special details

**Table:**

Geometry. All e.s.d.'s (except the e.s.d. in the dihedral angle between two l.s. planes) are estimated using the full covariance matrix. The cell e.s.d.'s are taken into account individually in the estimation of e.s.d.'s in distances, angles and torsion angles; correlations between e.s.d.'s in cell parameters are only used when they are defined by crystal symmetry. An approximate (isotropic) treatment of cell e.s.d.'s is used for estimating e.s.d.'s involving l.s. planes.
Refinement. Refinement of *F*^2^ against ALL reflections. The weighted *R*-factor *wR* and goodness of fit *S* are based on *F*^2^, conventional *R*-factors *R* are based on *F*, with *F* set to zero for negative *F*^2^. The threshold expression of *F*^2^ > σ(*F*^2^) is used only for calculating *R*-factors(gt) *etc*. and is not relevant to the choice of reflections for refinement. *R*-factors based on *F*^2^ are statistically about twice as large as those based on *F*, and *R*- factors based on ALL data will be even larger.

### Fractional atomic coordinates and isotropic or equivalent isotropic displacement parameters (Å^2^)

**Table:**

	*x*	*y*	*z*	*U*_iso_*/*U*_eq_	
C1	−0.1534 (2)	0.24120 (16)	0.80993 (12)	0.0329 (3)	
C2	0.0697 (2)	0.30067 (16)	0.82279 (12)	0.0351 (3)	
C3	0.2653 (3)	0.6592 (2)	0.61457 (15)	0.0559 (4)	
H3	0.4206	0.5901	0.6257	0.067*	
C4	0.1280 (4)	0.6664 (2)	0.52534 (17)	0.0712 (6)	
H4	0.1910	0.6018	0.4770	0.085*	
C5	−0.1001 (4)	0.7681 (2)	0.50819 (16)	0.0685 (5)	
H5	−0.1925	0.7726	0.4484	0.082*	
C6	−0.1917 (3)	0.8628 (2)	0.57895 (16)	0.0605 (5)	
H6	−0.3468	0.9319	0.5669	0.073*	
C7	−0.0578 (3)	0.85767 (18)	0.66808 (14)	0.0463 (4)	
H7	−0.1221	0.9234	0.7154	0.056*	
C8	0.1728 (3)	0.75450 (16)	0.68720 (12)	0.0364 (3)	
C9	0.3166 (2)	0.74777 (15)	0.78093 (12)	0.0329 (3)	
C11	0.4589 (3)	0.81235 (18)	0.91542 (13)	0.0451 (4)	
H11	0.4762	0.8682	0.9645	0.054*	
C10	0.5995 (3)	0.67346 (17)	0.91074 (13)	0.0414 (4)	
H10	0.7336	0.6140	0.9556	0.050*	
N1	0.5092 (2)	0.63481 (12)	0.82744 (10)	0.0363 (3)	
H1	0.5683	0.5498	0.8079	0.044*	
N2	0.2843 (2)	0.85814 (13)	0.83489 (11)	0.0409 (3)	
H2	0.1713	0.9444	0.8211	0.049*	
O1	0.04670 (19)	0.40133 (14)	0.86821 (12)	0.0591 (3)	
O2	0.27912 (17)	0.22797 (13)	0.78035 (11)	0.0565 (3)	
H2A	0.3941	0.2626	0.7889	0.085*	
O3	−0.12709 (18)	0.09884 (11)	0.82754 (11)	0.0547 (3)	
O4	−0.35046 (16)	0.34460 (11)	0.78500 (9)	0.0420 (3)	

### Atomic displacement parameters (Å^2^)

**Table:**

	*U*^11^	*U*^22^	*U*^33^	*U*^12^	*U*^13^	*U*^23^
C1	0.0231 (6)	0.0340 (8)	0.0460 (8)	−0.0023 (6)	−0.0051 (6)	−0.0198 (6)
C2	0.0249 (7)	0.0331 (8)	0.0510 (9)	−0.0015 (6)	−0.0098 (6)	−0.0183 (7)
C3	0.0585 (11)	0.0547 (10)	0.0585 (10)	−0.0027 (8)	−0.0167 (8)	−0.0242 (8)
C4	0.0890 (15)	0.0786 (14)	0.0595 (11)	−0.0185 (12)	−0.0179 (11)	−0.0330 (10)
C5	0.0738 (14)	0.0881 (15)	0.0521 (11)	−0.0333 (12)	−0.0248 (10)	−0.0119 (10)
C6	0.0459 (10)	0.0726 (12)	0.0573 (10)	−0.0132 (9)	−0.0182 (8)	−0.0063 (9)
C7	0.0400 (8)	0.0490 (9)	0.0468 (9)	−0.0089 (7)	−0.0087 (7)	−0.0094 (7)
C8	0.0383 (8)	0.0332 (8)	0.0379 (8)	−0.0103 (6)	−0.0067 (6)	−0.0080 (6)
C9	0.0322 (7)	0.0257 (7)	0.0399 (8)	−0.0042 (6)	−0.0030 (6)	−0.0106 (6)
C11	0.0470 (9)	0.0474 (9)	0.0493 (9)	−0.0059 (7)	−0.0126 (7)	−0.0252 (7)
C10	0.0403 (8)	0.0394 (8)	0.0453 (8)	−0.0024 (7)	−0.0139 (7)	−0.0142 (7)
N1	0.0371 (6)	0.0279 (6)	0.0441 (7)	0.0010 (5)	−0.0094 (5)	−0.0148 (5)
N2	0.0380 (7)	0.0308 (6)	0.0551 (8)	0.0034 (5)	−0.0114 (6)	−0.0195 (6)
O1	0.0379 (6)	0.0655 (8)	0.0974 (9)	−0.0077 (5)	−0.0096 (6)	−0.0560 (7)
O2	0.0216 (5)	0.0618 (7)	0.1047 (9)	−0.0057 (5)	−0.0051 (5)	−0.0522 (7)
O3	0.0314 (5)	0.0331 (6)	0.1060 (9)	−0.0020 (4)	−0.0108 (6)	−0.0311 (6)
O4	0.0235 (5)	0.0358 (6)	0.0717 (7)	0.0009 (4)	−0.0142 (5)	−0.0241 (5)

### Geometric parameters (Å, °)

**Table:**

C1—O3	1.2291 (17)	C7—C8	1.385 (2)
C1—O4	1.2541 (16)	C7—H7	0.9300
C1—C2	1.531 (2)	C8—C9	1.455 (2)
C2—O1	1.1934 (17)	C9—N1	1.3293 (18)
C2—O2	1.3003 (16)	C9—N2	1.3374 (17)
C3—C8	1.384 (2)	C11—C10	1.329 (2)
C3—C4	1.385 (2)	C11—N2	1.366 (2)
C3—H3	0.9300	C11—H11	0.9300
C4—C5	1.368 (3)	C10—N1	1.3649 (19)
C4—H4	0.9300	C10—H10	0.9300
C5—C6	1.362 (3)	N1—H1	0.8600
C5—H5	0.9300	N2—H2	0.8600
C6—C7	1.378 (2)	O2—H2A	0.8200
C6—H6	0.9300		
			
O3—C1—O4	126.05 (12)	C8—C7—H7	120.0
O3—C1—C2	118.29 (11)	C3—C8—C7	118.80 (14)
O4—C1—C2	115.64 (12)	C3—C8—C9	120.56 (14)
O1—C2—O2	125.70 (13)	C7—C8—C9	120.64 (13)
O1—C2—C1	122.10 (12)	N1—C9—N2	106.32 (12)
O2—C2—C1	112.19 (12)	N1—C9—C8	127.46 (12)
C8—C3—C4	120.32 (17)	N2—C9—C8	126.19 (12)
C8—C3—H3	119.8	C10—C11—N2	107.50 (13)
C4—C3—H3	119.8	C10—C11—H11	126.3
C5—C4—C3	120.15 (18)	N2—C11—H11	126.3
C5—C4—H4	119.9	C11—C10—N1	106.80 (13)
C3—C4—H4	119.9	C11—C10—H10	126.6
C6—C5—C4	119.83 (17)	N1—C10—H10	126.6
C6—C5—H5	120.1	C9—N1—C10	110.07 (11)
C4—C5—H5	120.1	C9—N1—H1	125.0
C5—C6—C7	120.91 (17)	C10—N1—H1	125.0
C5—C6—H6	119.5	C9—N2—C11	109.30 (12)
C7—C6—H6	119.5	C9—N2—H2	125.3
C6—C7—C8	120.00 (16)	C11—N2—H2	125.3
C6—C7—H7	120.0	C2—O2—H2A	109.5

### Hydrogen-bond geometry (Å, °)

**Table:**

*D*—H···*A*	*D*—H	H···*A*	*D*···*A*	*D*—H···*A*
O2—H2A···O4^i^	0.82	1.76	2.5793 (19)	172
N2—H2···O3^ii^	0.86	1.90	2.732 (2)	164
N1—H1···O4^i^	0.86	1.93	2.777 (2)	169

Symmetry codes: (i) *x*+1, *y*, *z*; (ii) *x*, *y*+1, *z*.
